# USP30: Structure, Emerging Physiological Role, and Target Inhibition

**DOI:** 10.3389/fphar.2022.851654

**Published:** 2022-03-03

**Authors:** Feng Wang, Yu Gao, Lihui Zhou, Junhao Chen, Zhiyan Xie, Zifan Ye, Yanfeng Wang

**Affiliations:** Key Laboratory of Molecular Medicine and Biotherapy, School of Life Science, Beijing Institute of Technology, Beijing, China

**Keywords:** ubiquitin-specific protease 30, structure, regulation, physiological role, target inhibition

## Abstract

Ubiquitin-specific protease 30 (USP30) is a deubiquitinating enzyme (DUB) belonging to the USP subfamily, which was found localized in the mitochondrial outer membrane and peroxisomes owing to its unique transmembrane domain. Structural study revealed that USP30 employed a unique catalytic triad and molecular architecture to preferentially cleave the Lys6 linked ubiquitin chains. USP30 plays an essential role in several cellular events, such as the PINK1/Parkin-mediated mitophagy, pexophagy, BAX/BAK-dependent apoptosis, and IKKβ–USP30–ACLY-regulated lipogenesis/tumorigenesis, and is tightly regulated by post-translational modification including phosphorylation and mono-ubiquitination. Dysregulation of USP30 is associated with a range of physiological disorders, such as neurodegenerative disease, hepatocellular carcinoma, pulmonary disorders, and peroxisome biogenesis disorders. Nowadays, scientists and many biopharmaceutical companies are making much effort to explore USP30 inhibitors including natural compounds, phenylalanine derivatives, *N*-cyano pyrrolidines, benzosulphonamide, and other compounds. For the treatment of pulmonary disorders, the study in Mission Therapeutics of USP30 inhibitor is already in the pre-clinical stage. In this review, we will summarize the current knowledge of the structure, regulation, emerging physiological role, and target inhibition of USP30, hoping to prompt further investigation and understanding of it.

## Introduction

Ubiquitin (Ub) is a small 76 amino acid-containing protein tag, which is critical in the aspect of regulating the protein destiny in cells (Ciechanover, 2003; Schwartz and Hochstrasser, 2003). Ubiquitination and deubiquitination are enzymatically catalyzed reversible processes by which ubiquitin is covalently bound by E1–E2–E3 enzymes or cleaved from a targeted protein by deubiquitinating enzymes (DUBs) (Komander et al., 2009). The ubiquitination and deubiquitination processes are involved in the regulation of various cellular events, such as cell cycle, cell apoptosis, and DNA repair. The ubiquitin modification system has been implicated in the pathogenesis of many diseases including neurodegenerative disease, cancer, inflammation, and viral infections, showing potential values as a therapeutic target (Harrigan et al., 2018). Analyses of the human genome have identified more than 100 members of DUBs, which are divided into seven major subfamilies based on the sequence and structural similarities (Mevissen and Komander, 2017). Amongst this, the ubiquitin-specific protease (USP) subfamily of proteins gained emerging focus for targeted drug discovery.

Ubiquitin modification is also a key regulatory process in various organelles, such as the maintenance of mitochondrial dynamics (Ling and Jarvis, 2013; Youle and Narendra, 2011). Mitochondria are tightly regulated by the ubiquitination of many factors involved in the biogenesis, fusion, and fission of the dynamic organelles (Schmidt et al., 2021). Parkin is the known ubiquitin ligase which ubiquitinates several mitochondrial proteins (Youle and Narendra, 2011). Several DUBs have also been found to regulate mitochondrial homeostasis by antagonizing Parkin activity, including USP8 (Durcan et al., 2014), USP14 (Chakraborty et al., 2018), USP15 (Cornelissen et al., 2014), USP30 (Bingol et al., 2014), USP33 (Niu et al., 2020), and USP35 (Wang et al., 2015). Interference of these DUBs can lead to the enhancement of mitophagy, implicating a critical role in mitochondrial quality control. However, most of these DUBs such as USP14 were only examined at the level of cell line or *Drosophila* model, and evaluation of the role in higher animal models is yet to be conducted (Banerjee et al., 2020). USP30 is a key mitochondrial regulator and has been investigated in depth. Knockdown of USP30 was demonstrated both in the *Drosophila* and mice model, showing a potential role in antagonizing the Parkin-mediated mitophagy (Nakamura and Hirose, 2008; Cunningham et al., 2015).

USP30, a member of the USP subfamily, was found localized in the mitochondrial outer membrane and peroxisomes (Clague and Urbe, 2017; Nakamura and Hirose, 2008; Wang et al., 2015). USP30 preferentially cleaves Lys6-linked ubiquitin chains, distinct from most of the non-linkage-specific USP family DUBs (Cunningham et al., 2015). Recent structural progress on either human USP30 (hUSP30) or zebrafish USP30 (zUSP30) complexed with Lys6-linked di-ubiquitin (di-Ub) provided compelling evidence on the molecular mechanism for the preference cleavage of Lys6-linked ubiquitin chains by USP30 (Gersch et al., 2017; Sato et al., 2017). Additionally, USP30 is strictly regulated by post-translational modifications, such as phosphorylation and ubiquitination, to ensure its pivotal biological function (Bingol et al., 2014; Gu et al., 2021).

Studies demonstrated that USP30 opposes the mitophagy caused by the PINK1/Parkin-mediated cascade of ubiquitination and phosphorylation under the mitochondrial depolarization status (Bingol et al., 2014). Selective autophagy is an essential process to maintaining the abundance and health of cellular organelles. There is no exception to the peroxisomes. Under amino acid starvation conditions, the E3 ubiquitin ligase PEX2 enhances the peroxisome membrane protein ubiquitination leading to the pexophagy (Deosaran et al., 2013; Sargent et al., 2016). USP30 localized to peroxisomes and was shown to decrease pexophagy (Riccio et al., 2019). Dysregulation of pexophagy causes a group of autosomal recessive disorders including the best-characterized peroxisome biogenesis disorders (PBDs) (Braverman et al., 2016).

Mitochondrial dysfunctions are also linked to a large number of physiological disorders including neurodegenerative diseases, cancer, cardiovascular diseases, and metabolic disorders (Galluzzi et al., 2017; Schmidt et al., 2021). A study reported that depletion of USP30 enhances the clearance of mitochondria by increasing mitophagy and also promotes Parkin-mediated cell death. USP30 overexpression decreased PINK1/Parkin-mediated mitophagy in cells (Bingol et al., 2014). Moreover, depletion of USP30 sensitizes cancer cells to the drug ABT-737 via regulating the BAX/BAK-dependent apoptosis pathway, without needing the Parkin overexpression (Liang et al., 2015). Accordingly, inhibition of USP30 represents a potential actionable drug target for intervening the pathologies associated with PINK1/Parkin deficiency-induced mitophagy dysfunction, such as Parkinson’s disease and pulmonary fibrosis (Bingol and Sheng, 2016). Lately, scientists and many biopharmaceutical companies have made much effort in exploring USP30 inhibitors including natural compounds, phenylalanine derivatives, *N*-cyano pyrrolidines, benzosulphonamide, and other small molecules.

The physiological importance of USP30 remains to be disclosed. Recently, researchers have conducted studies to better understand USP30 from the molecular mechanism to pathophysiological role. In this review, we will summarize the molecular structure, and regulation of USP30, and the latest discoveries about USP30 in human diseases, such as hepatocellular carcinoma, pulmonary fibrosis, and neurodegenerative diseases. Meanwhile, we likewise summarize the development of inhibitors targeted on USP30, hoping to bring useful insight for understanding the function of USP30 and prompting the discovery of USP30 inhibitors for the treatment of diseases.

## Molecular Characteristics of USP30

### Structure of USP30

**FIGURE 1:**
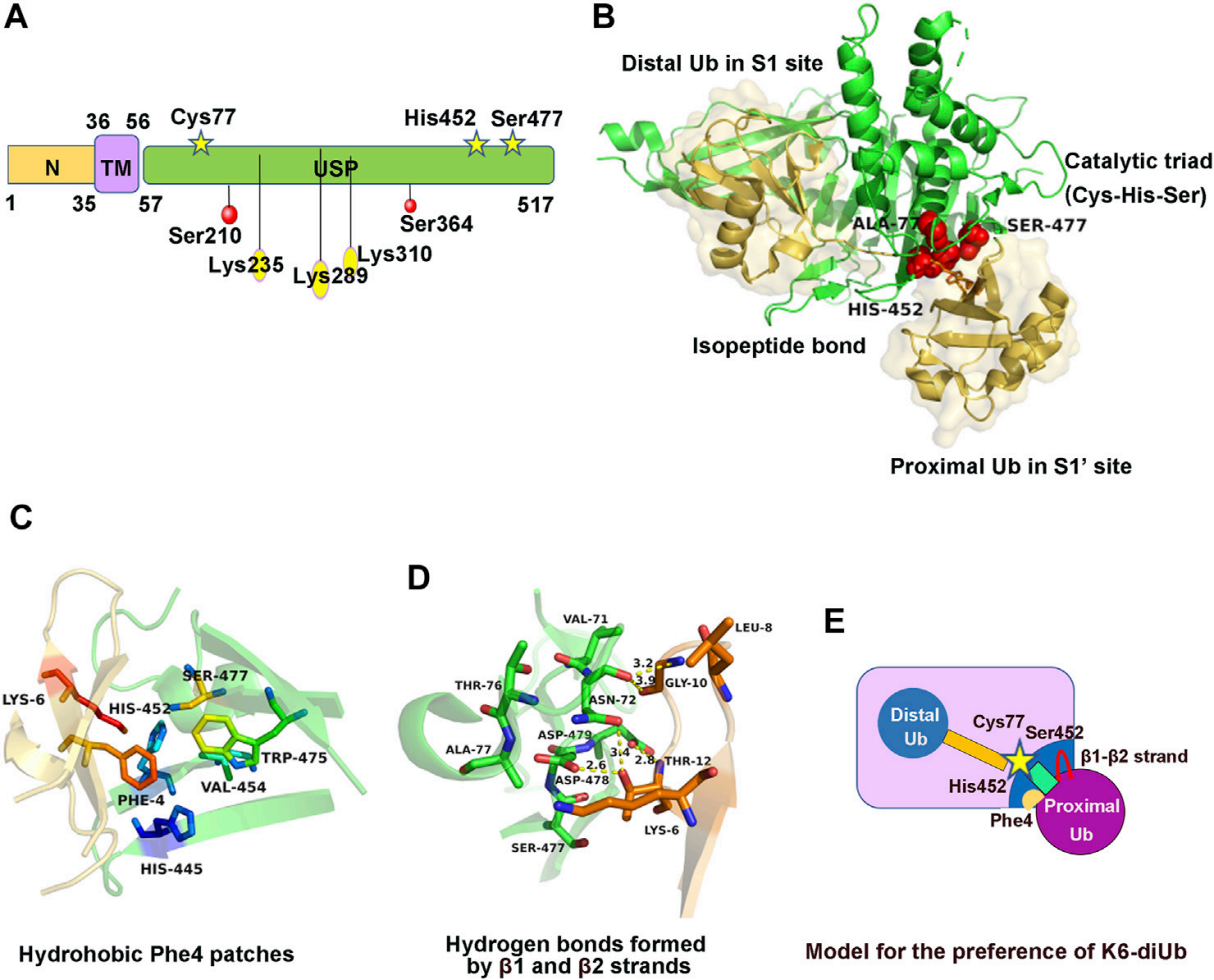
Structure, regulation, and molecular characteristics of USP30. **(A)** Domain structure and post-translational modification sites of USP30. **(B)** Crystal structure of USP30 complexed with K6-di-Ub. Ubiquitin molecules are shown with a transparent surface in light yellow. The catalytic triad is shown in red. **(C)** The ubiquitin Phe4 patches contacting a hydrophobic surface of USP30 near its catalytic triad. **(D)** Coordination of β1–β2 strands and USP30 thumb and palm domains to form hydrogen bonds. **(E)** Cartoon model to illustrate the preference of USP30 for the cleavage of K6-di-Ub.

USP30 was first identified in 2004 as the product of a gene 12q24.11, which contains 18 exons and mainly expressed in human skeletal muscle (Quesada et al., 2004). hUSP30 encodes a protein of 517 amino acids containing a mitochondrial targeting sequence and comprising a mitochondrial intermembrane domain (residues 1–35) in its N terminus, a transmembrane (TM) domain (residues 36–56), and a catalytic USP domain (residues 57–517) in its C terminus (Cunningham et al., 2015; Wauer et al., 2015). USP30 owns a unique catalytic triad consisting of Cys77, His452, and Ser477 which features an important serine residue as part of their catalytic triad (Ye et al., 2009) (Figure 1A). USP30 preferentially cleaves Lys6-linked ubiquitin chains (Cunningham et al., 2015), and recent structural progress on either the hUSP30 or zUSP30 complexed with Lys6-di-Ub revealed the molecular mechanism for their preference cleavage of Lys6-linked Ub chains (Gersch et al., 2017; Sato et al., 2017).

The crystal structure of the hUSP30–Lys6-di-Ub complex was obtained at 2.8Å resolution. Similar to the known structure of other USP family members, hUSP30 also comprises three subdomains named the thumb, palm, and fingers domain (Gersch et al., 2017; Sato et al., 2017). The distal Ub (Ub^dist^) contacts the S1 site including the thumb, fingers, and palm domain of USP30, whereas the proximal Ub (Ub^prox^) contacts the S1’ site including only the thumb and palm domain. The Ub^dist^-recognition mechanism is similar to that of other USP family proteins. Structural analysis showed the C-terminal tail of Ub^dist^ is stabilized by hydrophobic force and extensive hydrogen bond network. The proximal ubiquitin interaction is considerably weaker than the distal ubiquitin binding site, but the proximal binding site keeps the linkage preference of USP30 (Gersch et al., 2017; Sato et al., 2017) (Figure 1B). Three specific molecular features were observed in the USP30–di-Ub complex structure: first, the hydrophobic region of USP30 palm subdomain comprising three conserved residues His445, His452, and Trp475 that contact the ubiquitin Phe4 patches was not present in any other USP members (Figure 1C). Second, the ubiquitin β1 and β2 strands contact USP30 loops from the thumb and palm subdomains, forming several hydrogen bonds (Figure 1D). Third, the scissile isopeptide bond of Lys6-di-Ub was well located to the USP30 catalytic center and was fully engulfed by USP30 and stretches out the di-Ub, which adopts a distinct conformation from the compact structure in solution. Moreover, mutants of USP30 Ser477, His445, or Trp475 lost the Lys6-linkage preference to varying degrees (Gersch et al., 2017; Sato et al., 2017). Hence, the presence of the proximal ubiquitin binding site favored the Lys6-linkage preference of USP30 (Figure 1E).

### Regulation of USP30

USP30 plays an important role in various physiological processes and must be tightly regulated to keep its appropriate level and activity in biological events. Nevertheless, understanding of the regulation of USP30 is inadequate, particularly at the post-translational level (Wang and Wang, 2021). Recently, several known regulation ways of USP30 including phosphorylation and ubiquitination were identified, promoting the comprehension and development of appropriate therapeutic strategies.

Firstly, USP30 was phosphorylated in human hepatocellular carcinoma (HCC). Co-immunoprecipitation experiments conducted in HepG2 cell lines verified that the IκB kinase β (IKKβ) interacts with USP30 (Gu et al., 2021). Further study found that USP30 was directly phosphorylated on Ser210 and Ser364 sites by IKKβ, facilitating the stabilization of the USP30 (Gu et al., 2021). However, the effect of phosphorylation on USP30 activity remains unclear. Mutations of each site decreased the phosphorylation level of USP30, and the double Ser210 and Ser364 mutation significantly abolished its serine phosphorylation (Figure 1A). Moreover, IKKβ-induced USP30 phosphorylation and stabilization promote tumor growth and development in the dimethylnitrosamine (DEN)-/CCL4-induced model of HCC (Gu et al., 2021).

Secondly, USP30 can be ubiquitinated by the mitochondria ubiquitin ligase Parkin (Bingol et al., 2014). Since USP30 resides at the outer mitochondrial membrane and antagonizes the mitophagy process (Chan et al., 2011; Yoshii et al., 2011), it is reasonable to propose that the key mitophagy-related signaling molecule Parkin may be able to regulate USP30 (Cunningham et al., 2015). A study demonstrated that Parkin ubiquitinates USP30 both *in vivo* and *in vitro* (Gersch et al., 2017). In cells, Parkin ubiquitinates the endogenous USP30 and leads to its proteasome-dependent degradation, as MG132 inhibition can rescue the USP30 level (Bingol et al., 2014). *In vitro* reconstitution experiment revealed the mono-ubiquitination of USP30 on Lys235, Lys289, and Lys310 by the phosphorylated Parkin, matching the ubiquitination sites previously found in cells (Bingol et al., 2014; Cunningham et al., 2015; Gersch et al., 2017) (Figure 1A). Surprisingly, there was no significant difference in the activity between unmodified USP30 and monoubiquitinated USP30 in the ubiquitin-KG-TAMRA substrate cleavage assay (Gersch et al., 2017). Therefore, the impact of Parkin-mediated mono-ubiquitination of USP30 is unclear and still needs further study.

Furthermore, the key mitophagy signaling molecule PINK1 can regulate USP30 indirectly via phosphorylating the ubiquitin substrate (Wauer et al., 2015). As a protein kinase, PINK1 phosphorylates about 20% of the mitochondrial ubiquitin upon chemical depolarization to form Ser65-phosphorylated ubiquitin (Ordureau et al., 2015). Results indicated that USP30 has an ∼8-fold lower efficiency to hydrolyze the Ser65-phosphorylated ubiquitin-KG-TAMRA than to hydrolyze the unphosphorylated ubiquitin-KG-TAMRA. Moreover, the phosphorylated form of monoubiquitinated inactive USP30 weakened the deubiquitination by active USP30 (Gersch et al., 2017). Therefore, the phosphorylated ubiquitin is poor in the USP30 substrate, and PINK1 suppresses the function of USP30 indirectly by making the phosphorylated ubiquitin less susceptible to USP30 (Wauer et al., 2015). This finding can be illustrated structurally (Gersch et al., 2017). USP30 is an exo-DUB toward Lys6 linkages, and only unmodified Lys6-linked chains can interact with it (Mevissen and Komander, 2017). Thus, phosphorylation of the distal ubiquitin by PINK1 prevents the entire Lys6-linked chain from hydrolysis of USP30.

## Physiological Role of USP30

### USP30 in Neurodegenerative Disease

Neurodegenerative diseases are featured by the progressive damage to the nervous system such as the selective loss of neurons that leads to cognitive and motor behavior decline (Dugger and Dickson, 2017). Despite millions of people suffering from neurodegenerative disease worldwide, there are still no efficient drugs that intervene the neurodegenerative process to slow down or stop the disease progression (Schmidt et al., 2021). For example, the current therapeutics for Parkinson’s disease (PD) always alleviates the symptoms of parkinsonism at the early stage after diagnosis (Greenland and Barker, 2018). However, the treatment has become less effective with the advance of the neurodegenerative disease, and until now, there are no efficient therapies that block the progression of the disease (Miller and Muqit, 2019). Accordingly, it is urgent to understand the molecular basis of PD and other neurodegenerative diseases so that in the near future, breakthroughs can be made in the treatment of these diseases.

**FIGURE 2:**
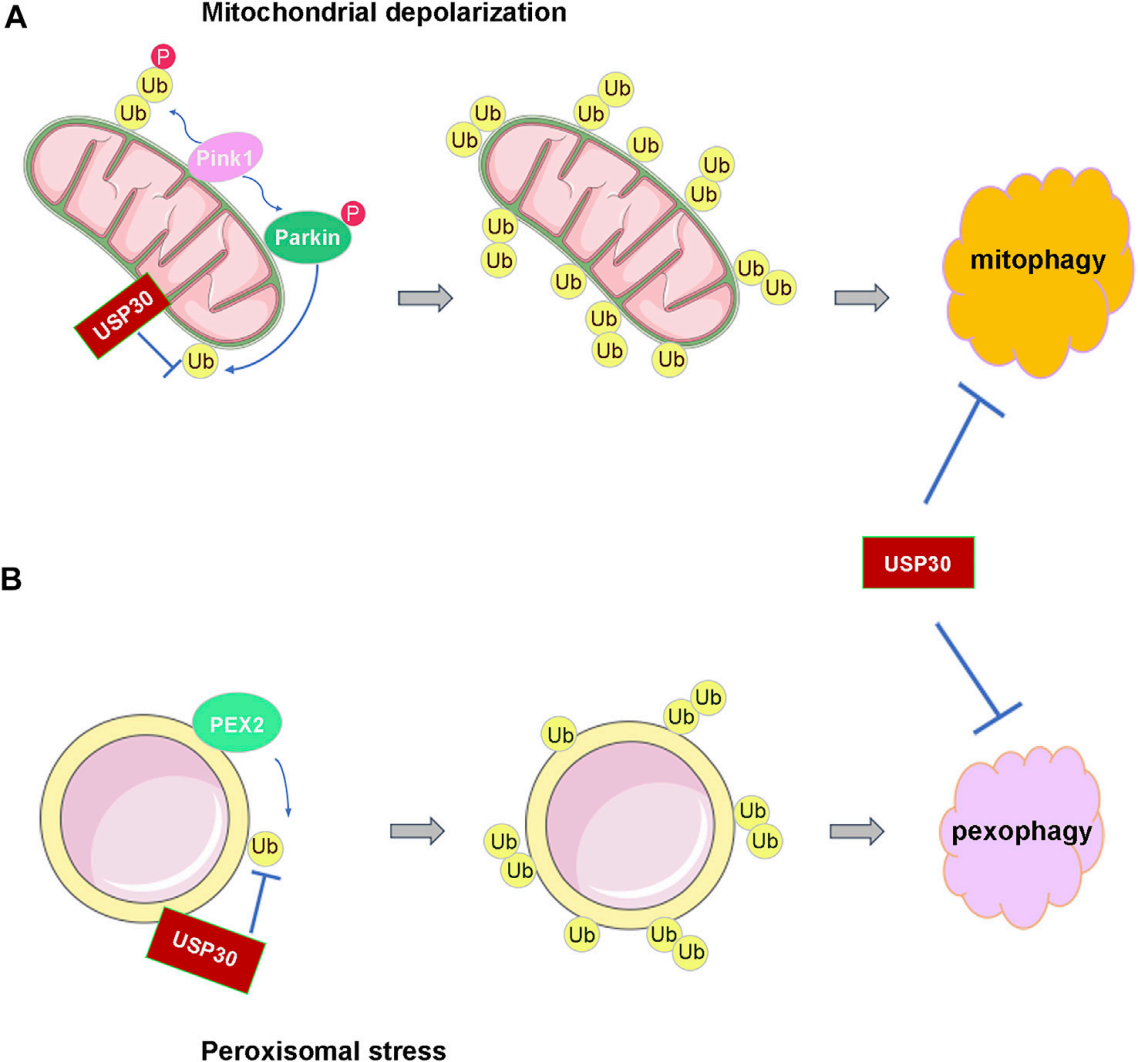
USP30 antagonizes the ubiquitination process of mitochondria and peroxisome to prevent their autophagy. **(A)** USP30 prevents the activation of Parkin and the ubiquitination of mitochondria and then antagonizes mitophagy under the depolarization condition. **(B)** USP30 also limits the pexophagy through reversing the ubiquitination of peroxisome by PEX2.

A characteristic of many neurodegenerative diseases is the misfolded protein such as β-amyloid (A β), α-synuclein, and huntingtin (htt) aggregates in different regions in the brain (Ciechanover and Kwon, 2015; Ross and Poirier, 2004). Additionally, many types of non-degradative ubiquitin signals are also essential for neuronal survival and functioning including mitochondrial homeostasis (Schmidt et al., 2021). Multiple lines of evidence demonstrated that mitochondrial dysfunction is critical to Parkinson’s disease (Ashrafi and Schwarz, 2013; Hauser and Hastings, 2013; Saiki et al., 2012). Mitochondrial dysfunction and reduced mitophagy are pathological hallmarks of both familial and sporadic PD (Bose and Beal, 2016; Luo et al., 2015; Osellame et al., 2013). The most well-characterized mitophagy pathway is the ubiquitin-dependent clearance of damaged mitochondria regulated by the mitochondrial-associated kinase PINK1 and the cytoplasmic E3 ubiquitin ligase Parkin (Kitada et al., 1998; Valente et al., 2004a). Upon mitochondrial depolarization, PINK1 stabilized on the outer membrane of the mitochondria where it dimerizes and autoactivates, then PINK1 phosphorylates the ubiquitin that conjugated to the mitochondrial protein, subsequently Parkin was recruited and phosphorylated by PINK1 which fully authorized its E3 ligase activity to ubiquitinate various mitochondrial proteins, and thereafter, mitophagy occurred (Okatsu et al., 2012; Okatsu et al., 2013; Ordureau et al., 2020) (Figure 2A). Loss of function mutation in both Parkin and PINK1 is known to lead to the autosomal recessive early-onset PD (EOPD) (Kitada et al., 1998; Valente et al., 2004b).

Research discovered that Parkin assembles Lys6, Lys11, and Lys63 ubiquitin chains on the mitochondria under damaging conditions, and USP30 has a strong preference for cleaving mitochondrially conjugated Lys6- and Lys11-linked ubiquitin chains (Cunningham et al., 2015; Narendra et al., 2008; Narendra et al., 2010). Therefore, USP30 prevents Parkin’s ability to drive mitophagy by removing the ubiquitin attached by Parkin on damaged mitochondria (Nakamura and Hirose, 2008) (Figure 2A). In cultured neurons, overexpression of USP30 blocks the Parkin-drived mitophagy, whereas knockdown USP30 enhances mitochondrial degradation (Bingol et al., 2014). Additionally, knockdown of USP30 rescues the defective mitophagy caused by pathogenic mutations in Parkin- or PINK1-deficient flies, and knockdown of USP30 in the dopaminergic neurons ameliorated the defects in motor and organismal survival of flies (Bingol et al., 2014) (Table 1). Furthermore, USP30 knockout mice are viable and born with Mendelian ratios with no gross histological phenotypes. Consistent with a previous study, the mitophagy was accelerated by 50% via examining the mitochondrial function in the cultured hippocampal neurons derived from the USP30 knockout mice (Phu et al., 2020). Therefore, USP30 inhibition is potentially beneficial for the treatment of PD via promoting mitophagy.

**TABLE 1:** Physiological role of USP30 in cancer and autophagy-related disorders.

Disorder type	Disease	Cellular effect	Mechanism/pathway	Reference
Autophagy-related disorders	Parkinson’s disease (PD)	Overexpression of USP30 blocks the Parkin-drived mitophagy; knockdown of USP30 enhances the mitochondrial degradation	USP30–PINK1/Parkin	Bingol et al. (2014); Nakamura and Hirose, (2008)
	Peroxisome biogenesis disorders (PBDs)	Overexpression of USP30 prevents pexophagy; deletion of USP30 induces pexophagy under basal condition	USP30–PEX2-mediated ubiquitination	Marcassa et al. (2018); Marcassa et al. (2019); Riccio et al. (2019)
	Pulmonary disorders/idiopathic pulmonary fibrosis (IPF)	USP30 inhibitors promote mitophagy in the lung fibrosis model	USP30–PINK1/Parkin	Kobayashi et al. (2016); Adnot et al. (2019)
Cancer	Hepatocellular carcinoma (HCC)	USP30 was most upregulated in HCCs mice; USP30 knockout mice had fewer tumor nodules and decreased tumor burden	IKKβ–USP30–ACLY	Bauer et al. (2005); Burke and Huff. (2017); Gu et al. (2021)
	Human osteosarcoma/human breast cancer	USP30 promotes the cell apoptosis of U2-OS and MCF7	USP30–BAX/BAK	Liang et al. (2015)
	Lung adenocarcinoma	Regulate cancer cell metastatic	Unknown	Birchmeier et al., 2003; Gentile et al. (2008); Buus et al. (2009)

### USP30 in Peroxisome Biogenesis Disorders (PBDs)

Peroxisomes are essential metabolic organelles in eukaryotic cells, playing a particularly vital role in lipid metabolism, reactive oxygen species (ROS) metabolism, and ether–phospholipid biosynthesis (Smith and Aitchison, 2013; Wanders and Waterham, 2006). Aberrant regulation of pexophagy breaks peroxisome homeostasis, thereby causing many human diseases, such as PBDs and neurodegenerative disorders including Alzheimer’s disease and amyotrophic lateral sclerosis (Trompier et al., 2014; Braverman et al., 2016; Islinger et al., 2018; Jo et al., 2020). The best-characterized peroxisomal diseases are PBDs, a group of autosomal recessive development disorders in which peroxisome is aberrant (Braverman et al., 2016).

Pexophagy is induced by various kinds of cellular stresses including hypoxia and starvation (Nordgren et al., 2013). Under amino acid starvation circumstance, the peroxisomal E3 ubiquitin ligase PEX2 upregulated, leading to increased ubiquitination of peroxisome membrane protein, followed by the pexophagy (Deosaran et al., 2013; Sargent et al., 2016). Similar to mitophagy, the peroxisomal-localized deubiquitinating enzyme USP30 regulates pexophagy tightly. A study demonstrated that USP30 prevents pexophagy by counteracting the E3 ubiquitin ligase activity of PEX2 (Marcassa et al., 2018; Wang et al., 2015) (Figure 2B). During amino acid starvation, overexpression of USP30 prevents pexophagy by counteracting PEX2-mediated ubiquitination of PMP70 and PEX5, while deletion of USP30 induces pexophagy under basal condition (Marcassa et al., 2018; Marcassa et al., 2019; Riccio et al., 2019). In the fibroblast cell line from patients with PEX1^G843D^ PBD (PEX1^G843D^, the most common PBD mutation), USP30 overexpression inhibits pexophagy by decreasing peroxisome ubiquitination and can recuse the loss of peroxisome (Braverman et al., 2013; Law et al., 2017; Riccio et al., 2019) (Table 1). Consequently, these studies may provide an exciting opportunity for PBD patients’ therapy by targeting pexophagy. Obviously, more work is still required to fully understand the mechanism of USP30-regulated pexophagy in PBDs.

### USP30 in Hepatocellular Carcinoma

The most famous and well-studied physiological function of USP30 is its role in mitophagy and the linkage to neurodegenerative disease. Nevertheless, no other report on the role of USP30 in tumorigenesis/lipogenesis or other physiological processes has been found. Cancer cells usually exhibit dysregulated lipid metabolism and inflammation (Currie et al., 2013). A recent study found that USP30 was most upregulated in both mRNA and protein level in HCC mice that sustained on high-fat diets (Gu et al., 2021). In HCC, the IκB kinase (IKKβ) phosphorylated and stabilized USP30, which promoted USP30 to deubiquitinate the critical lipogenesis-related enzyme-ATP citrate lyase (ACLY) (Bauer et al., 2005; Burke and Huff, 2017), and prompted the development of HCC (Gu et al., 2021). USP30 knockout mice had fewer tumor nodules and decreased tumor burden and largely attenuated the lipogenesis, inflammation, and hepatocarcinogenesis (Gu et al., 2021) (Table 1). Therefore, the study identified the axis of IKKβ–USP30–ACLY that plays a pivotal role in lipogenesis and liver cancer and may be a potential therapeutic target in the treatment of HCC.

### USP30 in Cell Death and Apoptosis

At the cellular level, several studies provide evidence for the pivotal role of USP30 involved in mitochondrial cell death and apoptosis. Mitochondria are fundamental in the orchestration of cell death pathways as they play a central role in energy production and metabolism (Martinou and Youle, 2011). USP30 can deubiquitinate the mitochondrial Parkin substrates and contributes to the orchestration of apoptotic cell death pathways (Liang et al., 2015). In Parkin-overexpressing human retinal pigment epithelial (hTERT-RPE1) cells, USP30 impedes Parkin-dependent ubiquitination of TOM20, and USP30 deletion promotes the depolarization-induced cell death (Liang et al., 2015). In addition, in human osteosarcoma (U2-OS) and human breast cancer (MCF7) cells, USP30 regulates the BAX/BAK-dependent apoptosis and its deletion sensitizes cancer cells to BH3-mimetics which can promote cell apoptosis (Liang et al., 2015). In lung adenocarcinoma cells, siRNA screen identified USP30 as one of the strongest hits involved in the hepatocyte growth factor (HGF)-induced cell scattering response (Birchmeier et al., 2003; Gentile et al., 2008), implying a role of USP30 in cancer cell metastatic (Buus et al., 2009) (Table 1). Putting these results together, it can be seen that USP30 may be a valuable target for combinatorial anti-cancer therapy.

### USP30 in Pulmonary Disorders

Mitophagy is principally governed by PINK1 and Parkin, a highly conserved mechanism of selectively clearing the damaged mitochondria for lysosomal degradation (Youle and Narendra, 2011; Vincow et al., 2013). Emerging evidence indicates that PINK1/Parkin-mediated mitophagy plays an essential role in the pathogenesis of various kinds of aging-associated pulmonary disorders, such as chronic obstructive pulmonary disease (COPD) and idiopathic pulmonary fibrosis (IPF) (Tsubouchi et al., 2018; Siekacz et al., 2021). Accumulation of damaged mitochondria in the bronchial epithelial cells has been observed in the COPD lungs (Hara et al., 2013). Also, insufficient mitophagy has also been proposed in the lung fibrosis development process during IPF pathogenesis (Araya et al., 2013b). PINK1/Parkin-mediated mitophagy has an important role in regulating cell fates, including cellular senescence, programmed cell death, and myofibroblast differentiation during the pathogenesis of COPD and IPF (Araya et al., 2013a; Kuwano et al., 2016). Thus, finding the modalities to control appropriate levels of PINK1/Parkin-mediated mitophagy activation may represent a potential therapeutic option to intervene the aging-associated pathogenesis of COPD and IPF.

USP30 is a unique mitochondria-positioned DUB, opposing the PINK1/Parkin-mediated mitophagy. Thus, inhibition of USP30 may represent an actionable target to correct the PINK1/Parkin defect-associated pathologies of pulmonary fibrosis (Kobayashi et al., 2016) (Table 1). USP30 inhibitors promoting mitophagy act with comparable efficiency in the lung fibrosis model to the pirfenidone which is a therapy approved in IPF treatment (Adnot et al., 2019). In addition, Mission Therapeutics is exploring USP30 inhibitors in the pre-clinical stage for the treatment of IPF and other mitochondrial disorders, indicating a potential druggable role of USP30 in IPF therapy (Harrigan et al., 2018).

### Development of Inhibitors Targeting on USP30

Recently, emerging physiological roles of USP30 in neurodegenerative diseases, cancer, and pulmonary disorders have been reported, and mechanistic studies indicated the therapeutic potential of USP30 inhibitors. Scientists and many biopharmaceutical companies such as Mission Therapeutics and Forma Therapeutics put much effort into finding the specific and potent small molecule inhibitors targeted on USP30 both covalently and non-covalently. Most of these tool compounds have been characterized in biochemical enzymatic assays using ubiquitin probes, and the cellular study is also reported for most USP30 inhibitors, whereas limited *in vivo* properties have been provided. Furthermore, some of these inhibitors are currently in the biological evaluation or pre-clinical development stage (Yue et al., 2014; Kluge et al., 2018; Rusilowicz-Jones et al., 2020b; Cabrera et al., 2021). In the following, we will summarize the research and patent reported on the discovery and development of USP30 inhibitors.

**FIGURE 3:**
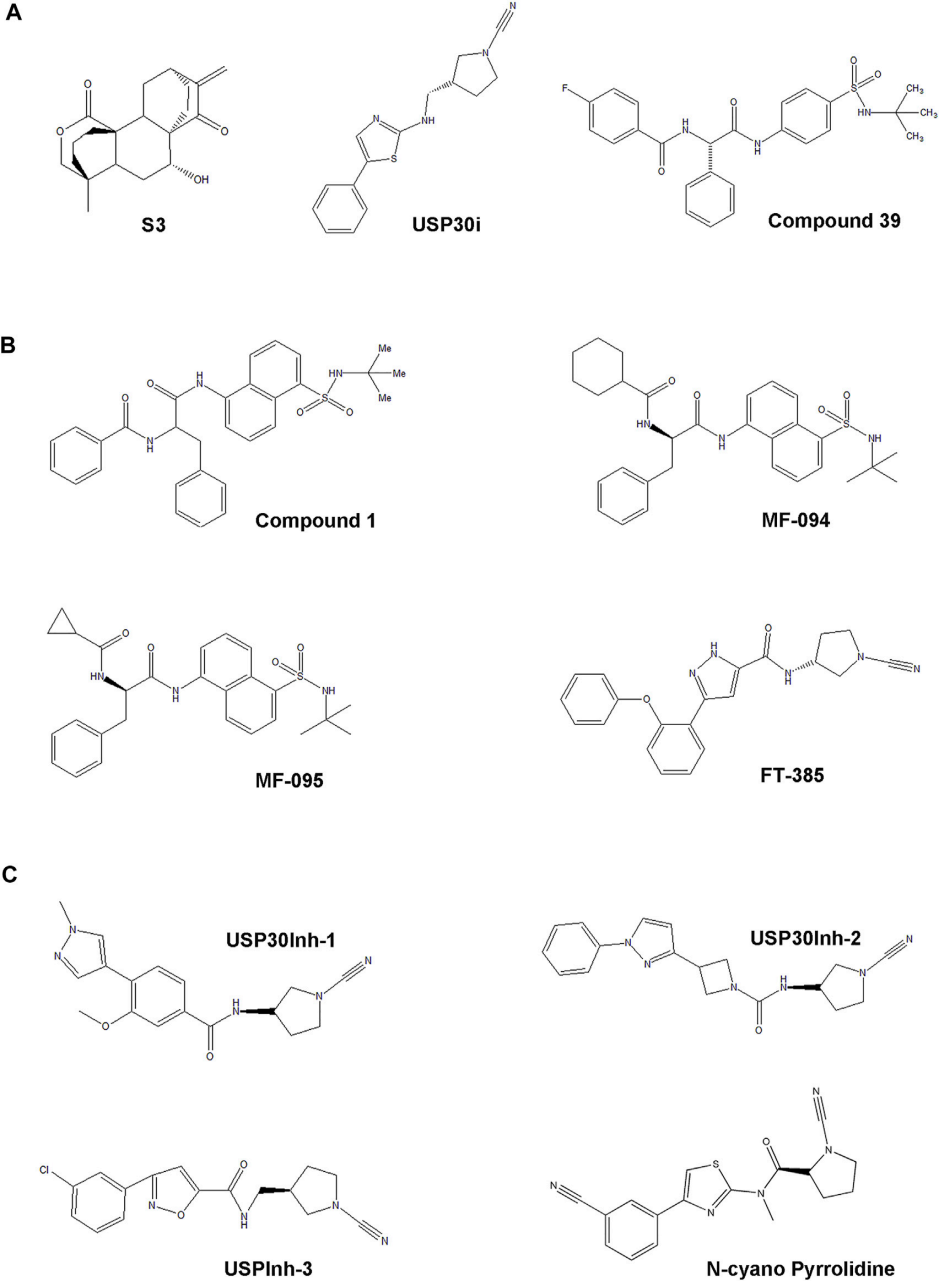
The development process and chemical structure of USP30 inhibitors**. (A)** The first small natural compound S3 was screened from 300 compounds in the cellular level. USP30i is a novel compound. Also, compound 39 is a benzosulphon amide compound characterized from a series of compounds reported in a previous study. **(B)** The racemic phenylalanine derivative compound 1 was identified from high-throughput screening, and MF-094, MF-095, and FT-385 were found based on the structure relationship study of the analogs derived from compound 1. **(C)** The cyano-amide containing small-molecule USP30Inh-1, USP30Inh-2, and USP30Inh-3 was synthesized based on the compound structure reported in a previous patent.

The first inhibitor, 15-oxospiramilactone (also named S3), a small natural diterpenoid derivative, was screened from 300 compounds based on the cellular level (Wang et al., 2011; Yue et al., 2014) (Figure 3A). 15-Oxospiramilactone could induce the elongation of mitochondria significantly in mitofusin1-deficient MEF cells. Cell lysates incubated with Biotin-S3 showed that S3 could directly inhibit the activity of USP30 via interacting with the cysteine77 residue in the catalytic triad (Yue et al., 2014). Previous studies found that high concentration of S3 induces apoptosis by inhibiting the Wnt pathway (Wang et al., 2011). Nonetheless, S3 does not lead to apoptosis at low concentration like 2μM, indicating a different mechanism from S3-induced mitochondrial fusion (Yue et al., 2014). Thus, the identification of S3 brings new implications for the treatment of these diseases related to dysfunction of mitochondrial dynamics (Table 2). However, additional investigation is still required to further elucidate the underlying molecular mechanism, specificity, and potency of S3.

**TABLE 2:** The development process and characterization of USP30 inhibitors.

Inhibitor type	Inhibitor	Development process	IC_50_	Inhibition mechanism	Specificity	Cellular effect	Reference
Small natural diterpenoid derivative	—	Screened from 300 compounds in the cellular level	Unknown	Covalent inhibitor, directly inhibit USP30 *via* interacting with the cysteine77 residue in the catalytic domain	High concentration of S3 inhibits the Wnt pathway	Induce the elongation of mitochondria in mitofusin1-deficient MEF cells	Wang et al., 2011; Yue et al. (2014)
Racemic phenylalanine derivative	Compound 1	High-throughput screen	<1 μM	Covalent modification	Relatively specific and does not inhibit USP1, USP8, and USP9 at concentration lower than 10 μM	Unknown	Kim et al., 2009; Dufner et al (2015); Paemka et al. (2015)
		Structure relationship (SAR) study of the analogs derived from compound 1	0.12 μM	Unknown	Have <30% inhibition activity for 22 ubiquitin-specific proteases up to 10 μM	MF-094 accelerated the mitophagy in C2C12 myotubes	Kluge et al. (2018)
	—		10 μM			No significant effect in C2C12 myotubes	
	FT385	Modified	∼1 nM	Covalent modification	Highly selective for USP30 up to 200nM, only USP6 showed a significant degree of inhibition	Recapitulate the promoting effects of USP30 depletion on mitophagy and show similar elevation of the ubiquitinated TOM20	Rusilowicz-Jones et al., 2020a
Cyano-amide containing small molecule	USP30Inh-1	Synthesized based on the compound structure reported in a previous patent	15–30 nM	Unknown	Show greatest off-target inhibition effect against USP6, USP21, and USP45	Inhibition of USP30 showed increased mitophagy in the SHSY5Y neuronal cells	Tsefou et al. (2021)
	USP30Inh-2						
	USP30Inh-3						
Novel compounds	USP30i	Unknown	2.45 μM	Unknown	Poor selectivity to DUB family members, such as UBP4, UBP45, and UBP47	Increase the ubiquitination of TOM20	Phu et al. (2020)
Benzosulphonamide	Compound 39	Characterized from a series of compounds reported in previous studies	∼20 nM	Unknown	Highly selective inhibition of compound 39 against USP30 without the off-target effect	Increased mitophagy and basal pexophagy	Kluge et al., 2018; Rusilowicz-Jones et al., 2021c

High-throughput screen identified a racemic phenylalanine derivative 1 (compound 1) as an USP30 inhibitor with an IC_50_ < 1 μM successfully by Mitobridge and Aurigene. Compound1 is relatively specific and does not inhibit USP1, USP8, and USP9 at concentration lower than 10 μM (Dufner et al., 2015; Kim et al., 2009; Paemka et al., 2015). Subsequently, Kluge et al. identified several potent and highly selective inhibitors of USP30 through the structure relationship (SAR) study of the analogs derived from compound 1 based on the enzymatic activity (Ub–Rho cleavage). Two analogs, MF-094 (compound 31) and MF-095 (compound 29), have <30% inhibition activity for 22 ubiquitin-specific protease under 10μM, showing good selectivity (Kluge et al., 2018) (Figure 3B). The IC_50_ of MF094 is 0.12μM, while MF095 has an IC_50_ higher than 10 μM. Two analogues MF-094 and MF095 inhibit USP30 with IC_50_ that are at least two orders of magnitude different. MF-094 was demonstrated to accelerate the mitophagy in C2C12 myotubes, while MF-095 did not lead to a significant effect (Kluge et al., 2018) (Table 2). Full characterization and *in vivo* study of these two compounds have not been reported. Notably, these are the first class of USP30 inhibitors that are expected to be non-covalent inhibitors without the *N*-cyano structural motif. Accordingly, the more potent MF-094 may represent a unique tool to explore the biological role of USP30 in the future.

The third class of USP30 inhibitors are some *N*-cyano pyrrolidines which have been reported in a panel of literature and patents (WO2016156816A1, WO2017009650A1, WO2017163078A1, WO2018060689A1, WO2018060691A1, WO2018060742A1, and WO2018065768A1 (Mission Therapeutics)). These structures are likely covalent inhibitors, which could form an adduct with the cysteine residues in protein via its *N*-cyano group, resembling the known Cathepsin C inhibitors (Laine et al., 2011). Some *N*-cyano pyrrolidines inhibitors were reported to be dual inhibitors of USP30 and UCHL1 in the earlier patent, and the selectivity and biological activity of the *N*-cyano pyrrolidines inhibitors still need to be investigated. Forma Therapeutics has also disclosed several patents describing compounds with N-cyano motifs and provided the ranges of inhibitory activity against USP30. Recently, Rusilowicz-Jones et al. identified a modified *N*-cyano pyrrolidines derivative FT385 inhibited USP30 with an IC_50_ of ∼1 nM (Rusilowicz-Jones et al., 2020a) (Figure 3B). Bio-layer interferometry experiments indicated FT385 inhibits USP30 through covalent modification. FT385 was highly selective for USP30 up to 200nM, and only USP6 exhibited a significant degree of inhibition (Urbe et al., 2012; Rusilowicz-Jones et al., 2020a). Cellular study shows that FT385 can recapitulate the promoting effects of USP30 depletion on mitophagy and show similar elevation of the ubiquitinated TOM20. Nevertheless, proteomics analyses conducted on the SHSY5Y neuroblastoma cell line with either genetic loss of USP30 or treated with FT385 disclosed some off-target inhibition of the drug (Rusilowicz-Jones et al., 2020a) (Table 2). Therefore, the development of specific inhibitors will make USP30 a more potential therapeutic target candidate in future.

Furthermore, Tsefou et al. synthesized three cyano-amide containing small molecule inhibitors (USP30Inh-1, USP30Inh-2, and USP30Inh-3) based on the compound structure reported in the previous patents (WO 2016/156,816 and WO 2017/103,614) (Figure 3C). USP30Inh-1, USP30Inh-2, and USP30Inh-3 all potently inhibit the activity of USP30 in cleavaging the Ub–Rho110 (ubiquitin–rhodamine 110) substrate with IC_50_ values between 15 and 30 nM (Tsefou et al., 2021). All three compounds show good selectivity against more than 40 known DUBs at 1 μM. Inhibition of USP30 showed increased mitophagy in the SHSY5Y neuronal cells. However, decreased selectivity was observed for each compound at 10 μM in cellular studies, showing greatest off-target inhibition effect against USP6, USP21, and USP45(Tsefou et al., 2021). Therefore, USP30 inhibitors containing the cyano-amide functional group have some off-target effects when using higher concentrations (Table 2). These studies emphasize the need to carefully profile the USP30 inhibitors in cellular studies in order to avoid the off-target effect.

Additionally, Kemp et al. published the USP30 inhibitor (USP30i) in the patent WO 2017103614 (Figure 3A). Subsequently, the cellular property of USP30i was characterized by Phu et al., in 2020 (Phu et al., 2020). By measuring the ubiquitination of TOM20 (a known USP30 substrate, Ub-TOM20), it was indicated that USP30i increased Ub-TOM20 with an EC_50_ of 2.45 μM. Then, the off-target effect of USP30i was detected by analyzing the ubiquitinome of USP30−/− HEK293T and the wild-type cells treated with USP30i (Phu et al., 2020). Among the off targets identified, many belonged to the DUB family, such as UBP4, UBP45, and UBP47, highlighting the poor selectivity of the inhibitor USP30i (Table 2).

In light of the limitations of these inhibitors, Rusilowicz-Jones et al. further characterized benzosulphonamide (compound 39) from a series of compounds reported in previous studies (Kluge et al., 2018; Rusilowicz-Jones et al., 2020a) (Figure 3A). *In vitro* assay of enzyme activity showed compound 39 has an IC_50_ of ∼20nM, representing a highly selective inhibitor of USP30 from 1 to 100M concentration. By comparing USP30−/− and compound 39-treated WT cell off-target assessment, results validate the highly selective inhibition of compound 39 against USP30 without the off-target effect (Rusilowicz-Jones et al., 2020a). Moreover, upon application of compound 39 to the SHSY5Y neuronal cultures for 24 h, samples showed increased mitophagy. Compound 39-treated U2OS cells also showed a strong increase in the basal pexophagy (Rusilowicz-Jones et al., 2020a) (Table 2). Therefore, the benzosulphonamide USP30 inhibitor compound 39 represents an important new class of tool compound with good potency and specificity for the enhancement of mitophagy and pexophagy, providing further encouragement for the pre-clinical study of these compounds.

## Conclusion and Perspectives

In summary, in recent years, emerging studies illustrated the role of USP30 from the molecular mechanism to its important physiological function. USP30 employed a unique catalytic triad (Cys77, His452 and Ser477) and molecular structure to preferential cleave Lys6 linked ubiquitin chains, which is different from the non-selectivity of other USP members. USP30 plays an essential role in PINK1/Parkin-mediated mitophagy, pexophagy, BAX/BAK-dependent apoptosis, and IKKβ–USP30–ACLY signaling pathway and is tightly regulated by post-translational modification including phosphorylation and mono-ubiquitination. Dysregulation of USP30 is associated with a range of physiological disorders, such as neurodegenerative disease, hepatocellular carcinoma, pulmonary disorders, and peroxisome biogenesis disorders. Although the detailed mechanism and physiological role of USP30 in diseases still need further investigation, current studies have already indicated the possibility of USP30 as a potential actionable drug target.

Nowadays, scientists and drug companies are making much effort to explore USP30 inhibitors including natural compounds, phenylalanine derivatives, *N*-cyano pyrrolidines, benzosulphonamide, and other compounds. For example, Mission Therapeutics has published several patent applications in describing USP30 inhibitors, trying to find the treatment for Parkinson disease and other mitochondrial disorders. For the treatment of pulmonary disorders, the study of USP30 inhibitor is already in the pre-clinical stage. However, current inhibitors still confront the limitations of poor potency and off-target effect at higher concentrations. Also, the structure of the USP30 inhibitor complex is lacking, seriously hindering the further development and optimization of the inhibitors. In addition, as USP30 also governs the mitochondrial protein import at the TOM (translocase of the outer membrane complex), suggesting inhibition of USP30 may have toxic effects. Hence, studies in aged USP30−/− mice will be imperative to understand the long-term effect of USP30 inhibition and will raise more confidence for researchers on the proposition that USP30 is a pharmacologically druggable target.

## Abbreviations

ACLY, ATP citrate lyase; COPD, chronic obstructive pulmonary disease; EOPD, early-onset PD; HCC, hepatocellular carcinoma; HGF, hepatocyte growth factor; IPF, idiopathic pulmonary fibrosis; IKKβ, IkB kinase β; m DEN, dimethylnitrosamine; PD, Parkinson’s disease; SAR, structure relationship; USP30, ubiquitin-specific protease 30; DUB, deubiquitinating enzyme; Ub, ubiquitin; USP, ubiquitin-specific protease; Di-Ub, di-ubiquitin; PBDs, peroxisome biogenesis disorders; Ub^dist^, distal Ub; Ub^prox^, proximal Ub.

## References

[B1] Adnot S. Lipskaia L. Bernard D. (2019). The STATus of STAT3 in Lung Cell Senescence. Am. J. Respir. Cel Mol Biol 61, 5–6. 10.1165/rcmb.2019-0013ED PMC6604214 30673511

[B2] Araya J. Hara H. Kuwano K. (2013a). Autophagy in the Pathogenesis of Pulmonary Disease. Intern. Med. 52, 2295–2303. 10.2169/internalmedicine.52.1118 24126389

[B3] Araya J. Kojima J. Takasaka N. Ito S. Fujii S. Hara H. (2013b). Insufficient Autophagy in Idiopathic Pulmonary Fibrosis. Am. J. Physiol. Lung Cel Mol Physiol 304, L56–L69. 10.1152/ajplung.00213.2012 23087019

[B4] Ashrafi G. Schwarz T. L. (2013). The Pathways of Mitophagy for Quality Control and Clearance of Mitochondria. Cell Death Differ 20, 31–42. 10.1038/cdd.2012.81 22743996 PMC3524633

[B5] Banerjee C. Roy M. Mondal R. Chakraborty J. (2020). USP14 as a Therapeutic Target against Neurodegeneration: A Rat Brain Perspective. Front Cel Dev Biol 8, 727. 10.3389/fcell.2020.00727 PMC7411183 32850842

[B6] Bauer D. E. Hatzivassiliou G. Zhao F. Andreadis C. Thompson C. B. (2005). ATP Citrate Lyase Is an Important Component of Cell Growth and Transformation. Oncogene 24, 6314–6322. 10.1038/sj.onc.1208773 16007201

[B7] Bingol B. Sheng M. (2016). Mechanisms of Mitophagy: PINK1, Parkin, USP30 and beyond. Free Radic. Biol. Med. 100, 210–222. 10.1016/j.freeradbiomed.2016.04.015 27094585

[B8] Bingol B. Tea J. S. Phu L. Reichelt M. Bakalarski C. E. Song Q. (2014). The Mitochondrial Deubiquitinase USP30 Opposes Parkin-Mediated Mitophagy. Nature 510, 370–375. 10.1038/nature13418 24896179

[B9] Birchmeier C. Birchmeier W. Gherardi E. Vande Woude G. F. (2003). Met, Metastasis, Motility and More. Nat. Rev. Mol. Cel Biol 4, 915–925. 10.1038/nrm1261 14685170

[B10] Bose A. Beal M. F. (2016). Mitochondrial Dysfunction in Parkinson's Disease. J. Neurochem. 139 Suppl 1 (Suppl. 1), 216–231. 10.1111/jnc.13731 27546335

[B11] Braverman N. E. D'Agostino M. D. Maclean G. E. (2013). Peroxisome Biogenesis Disorders: Biological, Clinical and Pathophysiological Perspectives. Dev. Disabil. Res. Rev. 17, 187–196. 10.1002/ddrr.1113 23798008

[B12] Braverman N. E. Raymond G. V. Rizzo W. B. Moser A. B. Wilkinson M. E. Stone E. M. (2016). Peroxisome Biogenesis Disorders in the Zellweger Spectrum: An Overview of Current Diagnosis, Clinical Manifestations, and Treatment Guidelines. Mol. Genet. Metab. 117, 313–321. 10.1016/j.ymgme.2015.12.009 26750748 PMC5214431

[B14] Burke A. C. Huff M. W. (2017). ATP-citrate Lyase: Genetics, Molecular Biology and Therapeutic Target for Dyslipidemia. Curr. Opin. Lipidol. 28, 193–200. 10.1097/MOL.0000000000000390 28059952

[B15] Buus R. Faronato M. Hammond D. E. Urbé S. Clague M. J. (2009). Deubiquitinase Activities Required for Hepatocyte Growth Factor-Induced Scattering of Epithelial Cells. Curr. Biol. 19, 1463–1466. 10.1016/j.cub.2009.07.040 19699092 PMC2764384

[B16] Cabrera S. F. Muratore M. E. Buijnsters P. (2021). The Intriguing Role of USP30 Inhibitors as Deubiquitinating Enzymes from the Patent Literature since 2013. Expert Opin. Ther. Pat. 10.1080/13543776.2022.2003780 34743664

[B17] Chakraborty J. von Stockum S. Marchesan E. Caicci F. Ferrari V. Rakovic A. (2018). USP14 Inhibition Corrects an *In Vivo* Model of Impaired Mitophagy. EMBO Mol. Med. 10, 9014. 10.15252/emmm.201809014 PMC6220287 30249595

[B18] Chan N. C. Salazar A. M. Pham A. H. Sweredoski M. J. Kolawa N. J. Graham R. L. (2011). Broad Activation of the Ubiquitin-Proteasome System by Parkin Is Critical for Mitophagy. Hum. Mol. Genet. 20, 1726–1737. 10.1093/hmg/ddr048 21296869 PMC3071670

[B19] Ciechanover A. Kwon Y. T. (2015). Degradation of Misfolded Proteins in Neurodegenerative Diseases: Therapeutic Targets and Strategies. Exp. Mol. Med. 47, e147. 10.1038/emm.2014.117 25766616 PMC4351408

[B20] Ciechanover A. (2003). The Ubiquitin Proteolytic System and Pathogenesis of Human Diseases: a Novel Platform for Mechanism-Based Drug Targeting. Biochem. Soc. Trans. 31, 474–481. 10.1042/bst0310474 12653666

[B21] Clague M. J. Urbé S. (2017). Integration of Cellular Ubiquitin and Membrane Traffic Systems: Focus on Deubiquitylases. Febs J. 284, 1753–1766. 10.1111/febs.14007 28064438 PMC5484354

[B22] Cornelissen T. Haddad D. Wauters F. Van Humbeeck C. Mandemakers W. Koentjoro B. (2014). The Deubiquitinase USP15 Antagonizes Parkin-Mediated Mitochondrial Ubiquitination and Mitophagy. Hum. Mol. Genet. 23, 5227–5242. 10.1093/hmg/ddu244 24852371 PMC7108632

[B23] Cunningham C. N. Baughman J. M. Phu L. Tea J. S. Yu C. Coons M. (2015). USP30 and Parkin Homeostatically Regulate Atypical Ubiquitin Chains on Mitochondria. Nat. Cel Biol 17, 160–169. 10.1038/ncb3097 25621951

[B24] Currie E. Schulze A. Zechner R. Walther T. C. Farese R. V. Jr (2013). Cellular Fatty Acid Metabolism and Cancer. Cell Metab 18, 153–161. 10.1016/j.cmet.2013.05.017 23791484 PMC3742569

[B25] Deosaran E. Larsen K. B. Hua R. Sargent G. Wang Y. Kim S. (2013). NBR1 Acts as an Autophagy Receptor for Peroxisomes. J. Cel Sci 126, 939–952. 10.1242/jcs.114819 23239026

[B26] Dufner A. Kisser A. Niendorf S. Basters A. Reissig S. Schönle A. (2015). The Ubiquitin-specific Protease USP8 Is Critical for the Development and Homeostasis of T Cells. Nat. Immunol. 16, 950–960. 10.1038/ni.3230 26214742

[B27] Dugger B. N. Dickson D. W. (2017). Pathology of Neurodegenerative Diseases. Cold Spring Harb Perspect. Biol 9, 028035. 10.1101/cshperspect.a028035 PMC5495060 28062563

[B28] Durcan T. M. Tang M. Y. Pérusse J. R. Dashti E. A. Aguileta M. A. McLelland G. L. (2014). USP8 Regulates Mitophagy by Removing K6-Linked Ubiquitin Conjugates from Parkin. Embo J. 33, 2473–2491. 10.15252/embj.201489729 25216678 PMC4283406

[B29] Galluzzi L. Bravo-San Pedro J. M. Levine B. Green D. R. Kroemer G. (2017). Pharmacological Modulation of Autophagy: Therapeutic Potential and Persisting Obstacles. Nat. Rev. Drug Discov. 16, 487–511. 10.1038/nrd.2017.22 I 28529316 PMC5713640

[B30] Gentile A. Trusolino L. Comoglio P. M. (2008). The Met Tyrosine Kinase Receptor in Development and Cancer. Cancer Metastasis Rev. 27, 85–94. 10.1007/s10555-007-9107-6 18175071

[B31] Gersch M. Gladkova C. Schubert A. F. Michel M. A. Maslen S. Komander D. (2017). Mechanism and Regulation of the Lys6-Selective Deubiquitinase USP30. Nat. Struct. Mol. Biol. 24, 920–930. 10.1038/nsmb.3475 28945249 PMC5757785

[B32] Greenland J. C. Barker R. A. (2018). “The Differential Diagnosis of Parkinson's Disease. In Parkinson's Disease,” in Pathogenesis and Clinical Aspects. Editors Stoker T. B. Greenland J. C..

[B33] Gu L. Zhu Y. Lin X. Lu B. Zhou X. Zhou F. (2021). The IKKβ-USP30-ACLY Axis Controls Lipogenesis and Tumorigenesis. Hepatology 73, 160–174. 10.1002/hep.31249 32221968

[B34] Hara H. Araya J. Ito S. Kobayashi K. Takasaka N. Yoshii Y. (2013). Mitochondrial Fragmentation in Cigarette Smoke-Induced Bronchial Epithelial Cell Senescence. Am. J. Physiol. Lung Cel Mol Physiol 305, L737–L746. 10.1152/ajplung.00146.2013 24056969

[B35] Harrigan J. A. Jacq X. Martin N. M. Jackson S. P. (2018). Deubiquitylating Enzymes and Drug Discovery: Emerging Opportunities. Nat. Rev. Drug Discov. 17, 57–78. 10.1038/nrd.2017.152 28959952 PMC7097658

[B36] Hauser D. N. Hastings T. G. (2013). Mitochondrial Dysfunction and Oxidative Stress in Parkinson's Disease and Monogenic Parkinsonism. Neurobiol. Dis. 51, 35–42. 10.1016/j.nbd.2012.10.011 23064436 PMC3565564

[B37] Islinger M. Voelkl A. Fahimi H. D. Schrader M. (2018). The Peroxisome: an Update on Mysteries 2.0. Histochem. Cel Biol 150, 443–471. 10.1007/s00418-018-1722-5 PMC6182659 30219925

[B38] Jo D. S. Park N. Y. Cho D. H. (2020). Peroxisome Quality Control and Dysregulated Lipid Metabolism in Neurodegenerative Diseases. Exp. Mol. Med. 52, 1486–1495. 10.1038/s12276-020-00503-9 32917959 PMC8080768

[B39] Kim J. M. Parmar K. Huang M. Weinstock D. M. Ruit C. A. Kutok J. L. (2009). Inactivation of Murine Usp1 Results in Genomic Instability and a Fanconi Anemia Phenotype. Dev. Cel 16, 314–320. 10.1016/j.devcel.2009.01.001 PMC3134285 19217432

[B40] Kitada T. Asakawa S. Hattori N. Matsumine H. Yamamura Y. Minoshima S. (1998). Mutations in the Parkin Gene Cause Autosomal Recessive Juvenile Parkinsonism. Nature 392, 605–608. 10.1038/33416 9560156

[B41] Kluge A. F. Lagu B. R. Maiti P. Jaleel M. Webb M. Malhotra J. (2018). Novel Highly Selective Inhibitors of Ubiquitin Specific Protease 30 (USP30) Accelerate Mitophagy. Bioorg. Med. Chem. Lett. 28, 2655–2659. 10.1016/j.bmcl.2018.05.013 29935771

[B42] Kobayashi K. Araya J. Minagawa S. Hara H. Saito N. Kadota T. (2016). Involvement of PARK2-Mediated Mitophagy in Idiopathic Pulmonary Fibrosis Pathogenesis. J. Immunol. 197, 504–516. 10.4049/jimmunol.1600265 27279371

[B43] Komander D. Clague M. J. Urbé S. (2009). Breaking the Chains: Structure and Function of the Deubiquitinases. Nat. Rev. Mol. Cel Biol 10, 550–563. 10.1038/nrm2731 19626045

[B44] Kuwano K. Araya J. Hara H. Minagawa S. Takasaka N. Ito S. (2016). Cellular Senescence and Autophagy in the Pathogenesis of Chronic Obstructive Pulmonary Disease (COPD) and Idiopathic Pulmonary Fibrosis (IPF). Respir. Investig. 54, 397–406. 10.1016/j.resinv.2016.03.010 27886850

[B45] Lainé D. Palovich M. McCleland B. Petitjean E. Delhom I. Xie H. (2011). Discovery of Novel Cyanamide-Based Inhibitors of Cathepsin C. ACS Med. Chem. Lett. 2, 142–147. 10.1021/ml100212k 24900293 PMC4018094

[B46] Law K. B. Bronte-Tinkew D. Di Pietro E. Snowden A. Jones R. O. Moser A. (2017). The Peroxisomal AAA ATPase Complex Prevents Pexophagy and Development of Peroxisome Biogenesis Disorders. Autophagy 13, 868–884. 10.1080/15548627.2017.1291470 28521612 PMC5446072

[B47] Liang J. R. Martinez A. Lane J. D. Mayor U. Clague M. J. Urbé S. (2015). USP30 Deubiquitylates Mitochondrial Parkin Substrates and Restricts Apoptotic Cell Death. EMBO Rep. 16, 618–627. 10.15252/embr.201439820 25739811 PMC4428036

[B48] Ling Q. Jarvis P. (2013). Dynamic Regulation of Endosymbiotic Organelles by Ubiquitination. Trends Cel Biol 23, 399–408. 10.1016/j.tcb.2013.04.008 23706390

[B49] Luo Y. Hoffer A. Hoffer B. Qi X. (2015). Mitochondria: A Therapeutic Target for Parkinson's Disease. Int. J. Mol. Sci. 16, 20704–20730. 10.3390/ijms160920704 26340618 PMC4613227

[B50] Marcassa E. Kallinos A. Jardine J. Rusilowicz-Jones E. V. Clague M. J. Urbé S. (2019). New Aspects of USP30 Biology in the Regulation of Pexophagy. Autophagy 15, 1634–1637. 10.1080/15548627.2019.1615304 31356149 PMC6693448

[B51] Marcassa E. Kallinos A. Jardine J. Rusilowicz-Jones E. V. Martinez A. Kuehl S. (2018). Dual Role of USP30 in Controlling Basal Pexophagy and Mitophagy. EMBO Rep. 19, 595. 10.15252/embr.201745595 PMC6030704 29895712

[B52] Martinou J. C. Youle R. J. (2011). Mitochondria in Apoptosis: Bcl-2 Family Members and Mitochondrial Dynamics. Dev. Cel 21, 92–101. 10.1016/j.devcel.2011.06.017 PMC3156409 21763611

[B53] Mevissen T. E. T. Komander D. (2017). Mechanisms of Deubiquitinase Specificity and Regulation. Annu. Rev. Biochem. 86, 159–192. 10.1146/annurev-biochem-061516-044916 28498721

[B54] Miller S. Muqit M. M. K. (2019). Therapeutic Approaches to Enhance PINK1/Parkin Mediated Mitophagy for the Treatment of Parkinson's Disease. Neurosci. Lett. 705, 7–13. 10.1016/j.neulet.2019.04.029 30995519

[B55] Nakamura N. Hirose S. (2008). Regulation of Mitochondrial Morphology by USP30, a Deubiquitinating Enzyme Present in the Mitochondrial Outer Membrane. Mol. Biol. Cel 19, 1903–1911. 10.1091/mbc.E07-11-1103 PMC2366858 18287522

[B56] Narendra D. Tanaka A. Suen D. F. Youle R. J. (2008). Parkin Is Recruited Selectively to Impaired Mitochondria and Promotes Their Autophagy. J. Cel Biol 183, 795–803. 10.1083/jcb.200809125 I PMC2592826 19029340

[B57] Narendra D. P. Jin S. M. Tanaka A. Suen D. F. Gautier C. A. Shen J. (2010). PINK1 Is Selectively Stabilized on Impaired Mitochondria to Activate Parkin. Plos Biol. 8, e1000298. 10.1371/journal.pbio.1000298 20126261 PMC2811155

[B58] Niu K. Fang H. Chen Z. Zhu Y. Tan Q. Wei D. (2020). USP33 Deubiquitinates PRKN/parkin and Antagonizes its Role in Mitophagy. Autophagy 16, 724–734. 10.1080/15548627.2019.1656957 31432739 PMC7138199

[B59] Nordgren M. Wang B. Apanasets O. Fransen M. (2013). Peroxisome Degradation in Mammals: Mechanisms of Action, Recent Advances, and Perspectives. Front. Physiol. 4, 145. 10.3389/fphys.2013.00145 23785334 PMC3682127

[B60] Okatsu K. Oka T. Iguchi M. Imamura K. Kosako H. Tani N. (2012). PINK1 Autophosphorylation upon Membrane Potential Dissipation Is Essential for Parkin Recruitment to Damaged Mitochondria. Nat. Commun. 3, 1016. 10.1038/ncomms2016 22910362 PMC3432468

[B61] Okatsu K. Uno M. Koyano F. Go E. Kimura M. Oka T. (2013). A Dimeric PINK1-Containing Complex on Depolarized Mitochondria Stimulates Parkin Recruitment. J. Biol. Chem. 288, 36372–36384. 10.1074/jbc.M113.509653 24189060 PMC3868751

[B62] Ordureau A. Heo J. M. Duda D. M. Paulo J. A. Olszewski J. L. Yanishevski D. (2015). Defining Roles of PARKIN and Ubiquitin Phosphorylation by PINK1 in Mitochondrial Quality Control Using a Ubiquitin Replacement Strategy. Proc. Natl. Acad. Sci. U S A. 112, 6637–6642. 10.1073/pnas.1506593112 25969509 PMC4450373

[B63] Ordureau A. Paulo J. A. Zhang J. An H. Swatek K. N. Cannon J. R. (2020). Global Landscape and Dynamics of Parkin and USP30-dependent Ubiquitylomes in iNeurons during Mitophagic Signaling. Mol. Cel 77, 1124–e10. 10.1016/j.molcel.2019.11.013 PMC7098486 32142685

[B64] Osellame L. D. Rahim A. A. Hargreaves I. P. Gegg M. E. Richard-Londt A. Brandner S. (2013). Mitochondria and Quality Control Defects in a Mouse Model of Gaucher Disease-Llinks to Parkinson's Disease. Cel Metab 17, 941–953. 10.1016/j.cmet.2013.04.014 PMC3678026 23707074

[B65] Paemka L. Mahajan V. B. Ehaideb S. N. Skeie J. M. Tan M. C. Wu S. (2015). Seizures Are Regulated by Ubiquitin-specific Peptidase 9 X-Linked (USP9X), a De-ubiquitinase. Plos Genet. 11, e1005022. 10.1371/journal.pgen.1005022 25763846 PMC4357451

[B66] Phu L. Rose C. M. Tea J. S. Wall C. E. Verschueren E. Cheung T. K. (2020). Dynamic Regulation of Mitochondrial Import by the Ubiquitin System. Mol. Cel 77, 1107–e10. 10.1016/j.molcel.2020.02.012 32142684

[B67] Quesada V. Díaz-Perales A. Gutiérrez-Fernández A. Garabaya C. Cal S. López-Otín C. (2004). Cloning and Enzymatic Analysis of 22 Novel Human Ubiquitin-specific Proteases. Biochem. Biophys. Res. Commun. 314, 54–62. 10.1016/j.bbrc.2003.12.050 I 14715245

[B68] Riccio V. Demers N. Hua R. Vissa M. Cheng D. T. Strilchuk A. W. (2019). Deubiquitinating Enzyme USP30 Maintains Basal Peroxisome Abundance by Regulating Pexophagy. J. Cel Biol 218, 798–807. 10.1083/jcb.201804172 I PMC6400567 30700497

[B69] Ross C. A. Poirier M. A. (2004). Protein Aggregation and Neurodegenerative Disease. Nat. Med. 10, S10–S17. 10.1038/nm1066 15272267

[B70] Rusilowicz-Jones E. Jardine J. Kallinos A. Pinto-Fernandez A. Guenther F. Giurrandino M. (2020a). A Novel USP30 Inhibitor Recapitulates Genetic Loss of USP30 and Sets the Trigger for PINK1-PARKIN Amplification of Mitochondrial Ubiquitylation. 10.1101/2020.04.16.044206 PMC7362391 32636217

[B71] Rusilowicz-Jones E. V. Barone F. G. Lopes F. M. Stephen E. Mortiboys H. Urbé S. (2020c). Benchmarking a Highly Selective USP30 Inhibitor for Enhancement of Mitophagy and Pexophagy). 10.1101/2021.04.28.441730 PMC8645336 34844982

[B72] Rusilowicz-Jones E. V. Jardine J. Kallinos A. Pinto-Fernandez A. Guenther F. Giurrandino M. (2020b). USP30 Sets a Trigger Threshold for PINK1-PARKIN Amplification of Mitochondrial ubiquitylation. Life Sci. Alliance 3, e20000768. 10.26508/lsa.202000768 PMC7362391 32636217

[B73] Saiki S. Sato S. Hattori N. (2012). Molecular Pathogenesis of Parkinson's Disease: Update. J. Neurol. Neurosurg. Psychiatry 83, 430–436. 10.1136/jnnp-2011-301205 22138181

[B74] Sargent G. van Zutphen T. Shatseva T. Zhang L. Di Giovanni V. Bandsma R. (2016). PEX2 Is the E3 Ubiquitin Ligase Required for Pexophagy during Starvation. J. Cel Biol 214, 677–690. 10.1083/jcb.201511034 PMC5021090 27597759

[B75] Sato Y. Okatsu K. Saeki Y. Yamano K. Matsuda N. Kaiho A. (2017). Structural Basis for Specific Cleavage of Lys6-Linked Polyubiquitin Chains by USP30. Nat. Struct. Mol. Biol. 24, 911–919. 10.1038/nsmb.3469 28945247

[B76] Schmidt M. F. Gan Z. Y. Komander D. Dewson G. (2021). Ubiquitin Signalling in Neurodegeneration: Mechanisms and Therapeutic Opportunities. Cel Death Differ 28, 570–590. 10.1038/s41418-020-00706-7 PMC7862249 33414510

[B77] Schwartz D. C. Hochstrasser M. (2003). A Superfamily of Protein Tags: Ubiquitin, SUMO and Related Modifiers. Trends Biochem. Sci. 28, 321–328. 10.1016/S0968-0004(03)00113-0 12826404

[B78] Siekacz K. Piotrowski W. J. Iwański M. A. Górski P. Białas A. J. Jakovljevic V. (2021). The Role of Interaction between Mitochondria and the Extracellular Matrix in the Development of Idiopathic Pulmonary Fibrosis. Oxid Med. Cel Longev 2021, 9932442–9932512. 10.1155/2021/9932442 PMC8545566 34707784

[B79] Smith J. J. Aitchison J. D. (2013). Peroxisomes Take Shape. Nat. Rev. Mol. Cel Biol 14, 803–817. 10.1038/nrm3700 PMC4060825 24263361

[B81] Trompier D. Vejux A. Zarrouk A. Gondcaille C. Geillon F. Nury T. (2014). Brain Peroxisomes. Biochimie 98, 102–110. 10.1016/j.biochi.2013.09.009 I 24060512

[B82] Tsefou E. Walker A. S. Clark E. H. Hicks A. R. Luft C. Takeda K. (2021).Investigation of USP30 Inhibition to Enhance Parkin-Mediated Mitophagy: Tools and Approaches. 10.1101/2021.02.02.429344 PMC8718267 34704599

[B83] Tsubouchi K. Araya J. Kuwano K. (2018). PINK1-PARK2-mediated Mitophagy in COPD and IPF Pathogeneses. Inflamm. Regen. 38, 18. 10.1186/s41232-018-0077-6 30386443 PMC6199723

[B84] Urbé S. Liu H. Hayes S. D. Heride C. Rigden D. J. Clague M. J. (2012). Systematic Survey of Deubiquitinase Localization Identifies USP21 as a Regulator of Centrosome- and Microtubule-Associated Functions. Mol. Biol. Cel 23, 1095–1103. 10.1091/mbc.E11-08-0668 PMC3302736 22298430

[B85] Valente E. M. Abou-Sleiman P. M. Caputo V. Muqit M. M. Harvey K. Gispert S. (2004a). Hereditary Early-Onset Parkinson's Disease Caused by Mutations in PINK1. Science 304, 1158–1160. 10.1126/science.1096284 15087508

[B86] Valente E. M. Salvi S. Ialongo T. Marongiu R. Elia A. E. Caputo V. (2004b). PINK1 Mutations Are Associated with Sporadic Early-Onset Parkinsonism. Ann. Neurol. 56, 336–341. 10.1002/ana.20256 15349860

[B87] Vincow E. S. Merrihew G. Thomas R. E. Shulman N. J. Beyer R. P. MacCoss M. J. (2013). The PINK1-Parkin Pathway Promotes Both Mitophagy and Selective Respiratory Chain Turnover *In Vivo* . Proc. Natl. Acad. Sci. U S A. 110, 6400–6405. 10.1073/pnas.1221132110 23509287 PMC3631677

[B88] Wanders R. J. Waterham H. R. (2006). Biochemistry of Mammalian Peroxisomes Revisited. Annu. Rev. Biochem. 75, 295–332. 10.1146/annurev.biochem.74.082803.133329 I 16756494

[B89] Wang W. Liu H. Wang S. Hao X. Li L. (2011). A Diterpenoid Derivative 15-oxospiramilactone Inhibits Wnt/β-Catenin Signaling and colon Cancer Cell Tumorigenesis. Cell Res 21, 730–740. 10.1038/cr.2011.30 21321609 PMC3203668

[B90] Wang Y. Serricchio M. Jauregui M. Shanbhag R. Stoltz T. Di Paolo C. T. (2015). Deubiquitinating Enzymes Regulate PARK2-Mediated Mitophagy. Autophagy 11, 595–606. 10.1080/15548627.2015.1034408 25915564 PMC4502823

[B91] Wang Y. Wang F. (2021). Post-translational Modifications of Deubiquitinating Enzymes: Expanding the Ubiquitin Code. Front. Pharmacol. 12, 685011. 10.3389/fphar.2021.685011 34177595 PMC8224227

[B92] Wauer T. Swatek K. N. Wagstaff J. L. Gladkova C. Pruneda J. N. Michel M. A. (2015). Ubiquitin Ser65 Phosphorylation Affects Ubiquitin Structure, Chain Assembly and Hydrolysis. Embo J. 34, 307–325. 10.15252/embj.201489847 25527291 PMC4339119

[B93] Ye Y. Scheel H. Hofmann K. Komander D. (2009). Dissection of USP Catalytic Domains Reveals Five Common Insertion Points. Mol. Biosyst. 5, 1797–1808. 10.1039/b907669g 19734957

[B94] Yoshii S. R. Kishi C. Ishihara N. Mizushima N. (2011). Parkin Mediates Proteasome-dependent Protein Degradation and Rupture of the Outer Mitochondrial Membrane. J. Biol. Chem. 286, 19630–19640. 10.1074/jbc.M110.209338 21454557 PMC3103342

[B95] Youle R. J. Narendra D. P. (2011). Mechanisms of Mitophagy. Nat. Rev. Mol. Cel Biol 12, 9–14. 10.1038/nrm3028 PMC4780047 21179058

[B96] Yue W. Chen Z. Liu H. Yan C. Chen M. Feng D. (2014). A Small Natural Molecule Promotes Mitochondrial Fusion through Inhibition of the Deubiquitinase USP30. Cel Res 24, 482–496. 10.1038/cr.2014.20 PMC3975501 24513856

